# Drug reaction with eosinophilia and systemic symptoms (DRESS) syndrome in two young children: the importance of an early diagnosis

**DOI:** 10.1186/s13052-018-0535-4

**Published:** 2018-08-15

**Authors:** Massimo Luca Castellazzi, Susanna Esposito, Laura Elisabetta Claut, Valeria Daccò, Carla Colombo

**Affiliations:** 1 Cystic Fibrosis Unit, Fondazione IRCCS Ca’ Granda Ospedale Maggiore Policlinico, Università degli Studi di Milano, 20122 Milan, Italy; 2 Pediatric Clinic, Department of Surgical and Biomedical Sciences, Università degli Studi di Perugia, Piazza Menghini 1, 06132 Perugia, Italy

**Keywords:** Antibiotic reactions, Antibiotic therapy, Cutaneous adverse reactions, DRESS syndrome, Drug exposure

## Abstract

**Background:**

Drug reaction with eosinophilia and systemic symptoms (DRESS) syndrome is a serious life-treating condition characterized by skin eruption, fever, haematologic abnormalities, and multi-organ involvement that can be fatal if unrecognized, especially in patients with liver failure. Diagnosis may be difficult because it is rarely described in children and can mimic many different conditions.

**Case presentation:**

We report two cases of DRESS syndrome due to prolonged antibiotic treatment in young children in whom recovery occurred following different therapeutic approaches. A previously healthy 5-year-old boy had been receiving intravenous vancomycin for right wrist and left elbow osteomyelitis and developed DRESS syndrome on day 30. The patient achieved a complete resolution of all symptoms with pulse methylprednisolone followed by oral prednisone. A 4-year-old girl with cystic fibrosis, pancreatic insufficiency, chronic pulmonary colonization by Gram-positive bacteria admitted for pulmonary exacerbation was treated with intravenous piperacillin-tazobactam and tobramycin. After 14 days of treatment, she developed DRESS syndrome: antibiotic treatment was therefore stopped, and without any further therapy, a progressive resolution of the patient’s clinical features was observed within 7 days, while the normalization of laboratory abnormalities was achieved at 14 days.

**Conclusions:**

Our cases highlight that paediatricians should be aware of the clinical presentations of and therapeutic approaches for DRESS syndrome, especially in children receiving long-term antibiotic treatment. The removal of the offending drug is crucial and may be the only life-saving measure. In more aggressive cases, corticosteroid or other immunosuppressive drugs should be considered to achieve the best outcome.

## Background

Drug reaction with eosinophilia and systemic symptoms (DRESS) syndrome is a rare, life-threatening, drug-induced hypersensitivity reaction. Drug hypersensitivity reactions (DHR) are classified as immediate and non-immediate. Immediate DHRs include urticaria, angioedema and anaphylaxis and occur immediately or within the first 6 h after administration of the drug. Non-immediate DHRs tend to appear after many days of treatment, with a delayed T-cell-dependent type of allergic mechanism and DRESS syndrome is considered as one of this kind of reactions [1].

DRESS is characterized by fever, rash, lymphadenopathy, elevated liver enzyme levels, and leukocytosis with eosinophilia [2]. DRESS syndrome is an uncommon condition with an estimated incidence that varies between 1:1000 and 1:10,000 drug exposures [3]. Furthermore, its prevalence is higher in adults than in children; therefore, paediatricians may not be sufficiently aware of this condition [4].

Prompt recognition and adequate management of DRESS are crucial because its clinical manifestations can be severe, resulting in a mortality rate of 10% [2]. To increase the likelihood of this condition being recognized, the European Register of Severe Cutaneous Adverse Reactions (RegiSCAR) developed a scoring system based on clinical findings, the extent of affected skin, the type of organ involvement and the clinical course to classify DRESS syndrome as defined, probable or possible [5]. In this report, we describe two cases of DRESS syndrome secondary to prolonged antibiotic exposure in young children. Our aim is to highlight the possible clinical presentations of this condition, the diagnostic tools to recognize it and the therapeutic approaches used to treat it in paediatric patients.

## Case presentation

Tables 1 and 2 summarize the clinical and laboratory data for each of the two patients.

**Table 1:** Clinical and laboratory data of the two children with drug reaction with eosinophilia and systemic symptoms (DRESS) syndrome

	ADMISSION		DRESS SYNDROME ONSET		DIAGNOSIS OF DRESS SYNDROME		DISCHARGE	
	Case 1	Case 2	Case 1	Case 2	Case 1	Case 2	Case 1	Case 2
Laboratory data (normal value)	Day 1	Day 1	Day 26	Day 14	Day 30	Day 18	Day 40	Day 28
WBC (4800–12,100/μL)	16,650	5600	14,980	8320	26,280	12,140	12,760	7820
Lymphocytes (1500–16,500//μL)	3120	2040	3800	4500	6710	4200	3200	3320
Eosinophils (100–500//μL)	200	220	2330	30	5010	2940	480	440
CRP (< 0.5 mg/dL)	19.59	0.59	3.60	10.31	6.1	3.19	0.03	0.14
AST-ALT (5–36 U/L and 5–29 U/L)	23–22	31–28	34–27	402–62	55–132	1560–311	25–45	36–60
LDH (120–300 U/L)	198	276	779	3637	805	10,880	238	300
PT-aPTT (0.94–1.22 and 0.86–1.20)	Not performed	Not performed	1.28–1.10	Not performed	1.31–1.06	1.23–1.94	0.98–0.95	0.96–1.03
D-dimer (< 230 ng/mL)	Not performed	Not performed	1815	Not performed	2000	68,384	120	230

*WBC* white blood cells, *CRP* C-reactive protein, *AST* aspartate aminotransferase, *ALT* alanine aminotransferase, *LDH* lactate dehydrogenase, *PT* prothrombin time, *aPTT* activated partial thromboplastin time

**Table 2:** Results of the RegiSCAR scoring system used to diagnose drug reaction with eosinophilia and systemic symptoms (DRESS) syndrome in two children

Items	Patient 1	Score: patient 1	Patient 2	Score: patient 2
Fever ≥ 38.5 °C	Yes	0	Yes	0
Enlarged lymph nodes	Yes	1	Yes	1
Eosinophils ^a^	Yes	2	Yes	2
Atypical lymphocytes	No	0	No	0
Skin rash > 50% of body surface area	Yes	1	Yes	1
Skin rash suggesting DRESS	Yes	1	Yes	1
Skin biopsy suggesting DRESS	Yes	1	Not applicable	0
Liver involvement	Yes	1	Yes	1
Resolution ≥ 15 days	No	-1	No	-1
Evaluation other potential causes ^b^	Negative	1	Negative	1
Total score		7		6

^a^: eosinophils 0.7–1.49 × 10^3^/mmc = 1; ≥1.5 × 10^3^/mmc = 2

^b^: include ANA, blood culture, serology for HVA/ HVB/ HVC, Chlamydia/ Mycoplasma pneumonia, other serology/PCR. None positive and ≥ 3 of the above negative = 1

Legend: Final score < 2: no case, final score 2–3: possible case, final score 4–5: probable case, and final score > 5: definite case

### Case 1

**Fig. 1:**
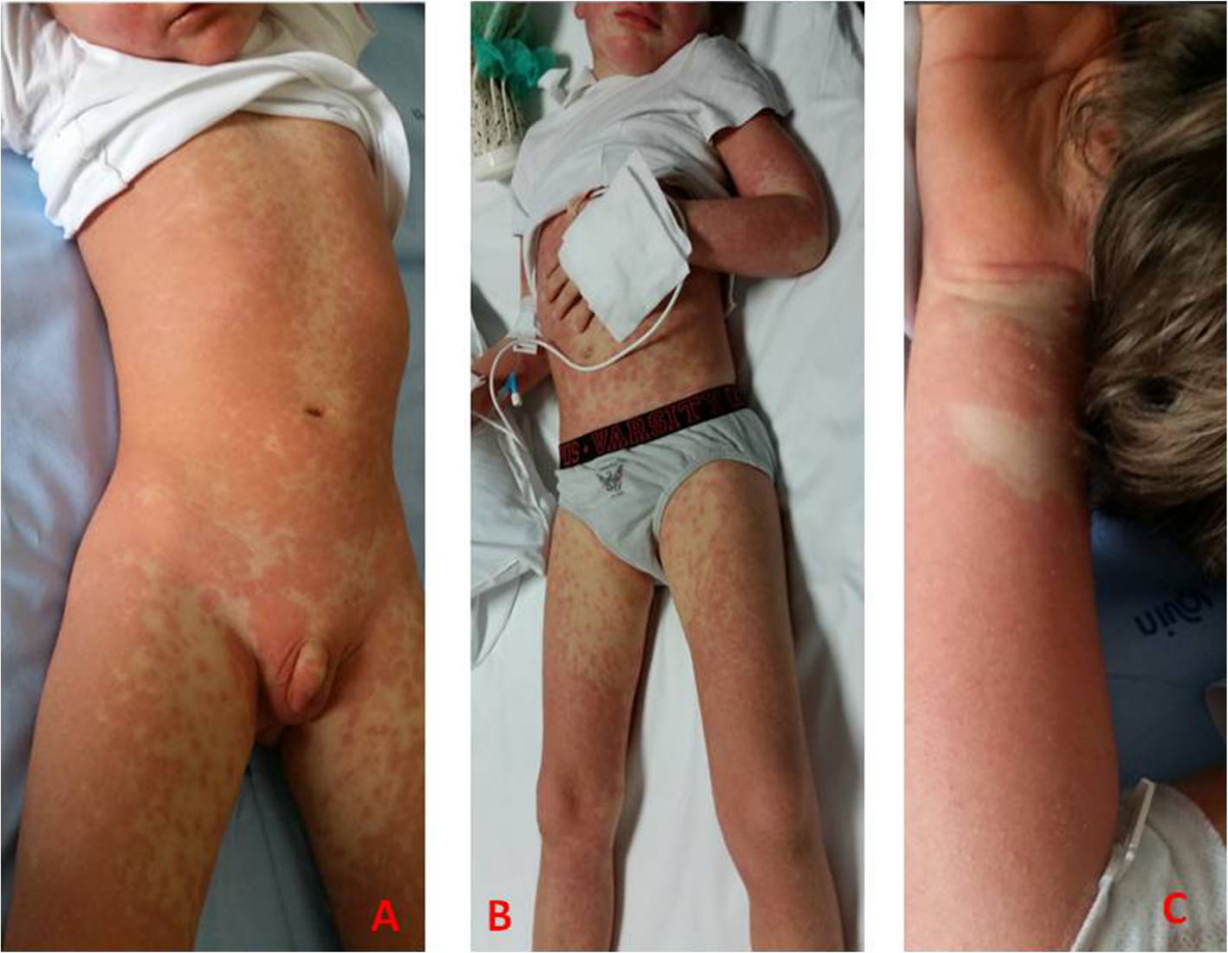
Patient 1 with a diffuse maculopapular erythaematous rash involving the face, trunk, back, penis and scrotum (panel **a**), legs (panel **b**), and arms (panel **c**)

A previously healthy 5-year-old boy had been receiving intravenous vancomycin (40 mg/kg/day four times per day) for 26 days due to right wrist and left elbow osteomyelitis. The patient had exhibited clinical and laboratory improvement. He then suddenly developed a generalized erythaematous maculopapular and pruritic rash involving the face, trunk, back and limbs followed by the appearance of a high-grade fever (up to 40 °C) and weakness. Bilateral cervical and inguinal enlarged lymph nodes were detected. Cardio-respiratory and abdominal examinations were normal. The child also developed facial, neck and scrotal oedema (Fig. 1).

Laboratory investigations revealed progressive leukocytosis (26,280/μL, normal value: 4800 − 12,100/ μL) and eosinophilia (5010/ μL; normal value: 100–500/ μL) on day 30. Liver function tests showed minimal alterations. In addition, lactate dehydrogenase (LDH) levels increased to 805 U/L (normal value: 120–300 U/lL. C-reactive protein (CRP) levels were slightly increased (6.10 mg/dL, normal value: < 0.5 mg/dL). Coagulation tests also showed alterations and a prolonged international normalized ratio (INR: 1.28, normal value: 0.94–1.22) and an increased d-dimer (1815 ng/mL, normal value: < 230 ng/ml). Renal function and electrolytes were normal. Virological examinations (including polymerase chain reaction for Epstein-Barr virus, cytomegalovirus, herpes-simplex virus, hepatitis and parvovirus) and autoimmune screening (anti-nuclear, anti-DNA, anti-neutrophil cytoplasmic, anti-smooth muscle, extractable nuclear antigen and anti-mitochondrial antibodies) were all negative.

A bone marrow aspirate did not show abnormalities, while a skin biopsy confirmed the presence of eosinophilic infiltration. Based on the patient’s clinical history and laboratory findings, the RegiSCAR scoring system was applied, and the boy was diagnosed with DRESS syndrome (total score = 7) on day 30. Vancomycin administration was discontinued and switched to oral linezolid (10 mg/kg/dose three times per day), which was interrupted 3 days later as a result of a worsening of the skin rash and the patient’s general condition. Thus, pulse methylprednisolone (20 mg/kg/day for 3 days) was started, resulting in rapid defervescence and prompt remission of the rash and facial-neck and scrotal edema within a few days. Oral prednisone (1.5 mg/kg/day) was continued, and the patient achieved a complete resolution of all symptoms and normal laboratory tests within 10 days. Prednisone was gradually reduced and finally discontinued after 1 month.

### Case 2

A 4-year-old girl with cystic fibrosis, pancreatic insufficiency and chronic pulmonary colonization by Gram-positive bacteria was admitted to our hospital for pulmonary exacerbation. Based on the last available sputum culture, intravenous piperacillin-tazobactam (150 mg/kg/day in three doses) and tobramycin (10 mg/kg in one dose) were started and resulted in progressive clinical improvement. Daily treatment with physiotherapy, an inhaled long-acting beta-agonist and oral pancreatic enzymes was continued throughout the patient’s hospitalization. After 14 days of treatment, she presented a high-grade fever (up to 40 °C) and a diffuse maculopapular erythaematous rash involving the trunk and eventually the whole body. She also developed generalized polyadenomegaly as well as hepatomegaly. Laboratory investigations showed a rise in CRP levels (10.31 mg/dL, normal value: < 0.5 mg/dL) and a progressive increase in serum transaminase levels, with aspartate aminotransferase and alanine aminotransferase levels > 40 U/L and > 10 times the upper limit of normal, respectively. Coagulation tests showed very high d-dimer concentrations (68,340 ng/mL, normal value: < 230 ng/mL), a prolonged activated partial thromboplastin time ratio (1.94, normal value: 0.86–1.20) and an INR of 1.23 (normal value: 0.94–1.22). LDH concentrations increased to 10,880 U/L at 4 days after the onset of symptoms. Also in this case, autoimmune, infective and haematologic tests were negative.

A parallel progressive increase in the patient’s eosinophil count reached a maximum absolute value of 2940/mmc on the 18th day. A diagnosis of DRESS syndrome was established based on a RegiSCAR total score of 6. Antibiotic treatment was therefore stopped, and without any further therapy, a progressive resolution of the patient’s clinical features was observed within 7 days, while the normalization of laboratory abnormalities was achieved at 14 days following the onset of DRESS syndrome (the 28th hospitalization day overall).

## Discussion and conclusions

DRESS syndrome is a rare, severe, drug-induced reaction characterized by a spectrum of systemic manifestations and multiple organ involvement. Common pharmacologic triggers for DRESS include aromatic anticonvulsants (mainly phenobarbital, phenytoin, and carbamazepine), antibiotics (mainly trimethoprim-sulfamethoxazole, minocycline, vancomycin, and anti-tubercular drugs), dapsone, allopurinol and nevirapine [6]. However, the list of drugs that have been associated with the development of DRESS syndrome is becoming longer and now includes ibuprofen, acetylsalicylic acid, sulthiame, and griseofulvin as possible triggers in children [7–10]. The aetiology of DRESS syndrome is not yet clear, but it has been suggested that this condition is multifactorial and may include an immune-mediated hypersensitivity component that is a direct effect of an interaction between the drugs or their metabolites and a genetic susceptibility [4]. Furthermore, an interplay between drugs, viruses (mainly herpes virus 6 [HHV6], but also HHV7, Epstein-Barr virus and cytomegalovirus) and immune system may have a role as trigger of DRESS syndrome [11]. In particular, it was observed that in patients with DRESS and HHV6 reactivation there was a higher levels of serum thymus and activation-regulated chemokine (TARC) that would lead to a Th2-type immune reaction [12]. Moreover, serum TARC were identified as a marker of severity of inflammation in drug eruptions [13].

DRESS syndrome typically manifests 2–6 weeks after the beginning of the administration of the offending drug [14]. However, early onset at 5 days has been described [15]. Interestingly, in a recent perspective study, children treated with antibiotics developed DRESS syndrome after an average latency of 5.8 days [16]. Fever usually precedes cutaneous eruption, which generally presents as a diffuse, pruritic, and macular rash [6, 14]. Furthermore, multiple organ systems may be involved. Lymphadenopathy is frequently described as similar to liver involvement and may progress to liver failure, which is the primary cause of death in DRESS syndrome [3]. Other systemic involvements include the kidneys, gastrointestinal tract, lungs, heart and central nervous system. Laboratory abnormalities associated with this condition include leukocytosis with peripheral eosinophilia, lymphocytosis and thrombocytopenia. Liver and renal function test results may also be altered [3, 14]. Because of its highly variable clinical presentation, other clinical conditions, such as acute viral infections, hepatitis, sepsis, autoimmune disease, and haematologic disorders, should be considered in the differential diagnosis of DRESS syndrome. In our patients, all the typical signs and symptoms of this condition (fever ≥38.5 °C, a skin rash extending over more than 50% of the body surface and lymphadenopathy) developed more than 14 days after the initiation of intravenous antibiotic therapy. Furthermore, both patients progressively presented typical biochemical abnormalities (eosinophilia and liver involvement). Atypical lymphocytes were not detected in our patients, and a skin biopsy was performed in only the first case. After other potential causes (autoimmune, infective and haematologic disorders) were excluded, the RegiSCAR scoring system was used to achieve a definite diagnosis of DRESS syndrome (total scores of 7 and 6 in cases 1 and 2, respectively; see Table 2).

To treat DRESS syndrome, the offending drug must be promptly removed. This may be sufficient to achieve the resolution of clinical and laboratory abnormalities, as we found in our second case. The pharmacological approach to treating this syndrome is not completely defined as such treatments have not yet been evaluated in clinical trials. Intravenous corticosteroids, administered alone or followed by oral steroid therapy, have been shown to be an effective treatment for DRESS syndrome [14, 17, 18]. However, there is no consensus regarding the dose and route of administration [19]. In our first case, considering the worsening of the skin rash and the general condition of the patient after the introduction of linezolid, pulse methylprednisolone was immediately administered, resulting in a rapid clinical improvement. In second patient, a progressive spontaneous resolution of the clinical features was observed within 7 days, while the normalization of laboratory abnormalities was achieved at 14 days following the onset of DRESS syndrome, highlighting the importance of an early diagnosis to avoid unfavourable outcome.

Of note, different reports have demonstrated an association of DRESS syndrome with subsequent autoimmune diseases (i.e., Graves disease, Hashimoto’s disease, type 1 diabetes mellitus, and autoimmune hemolytic anemia) [20, 21]. A gradual tapering of corticosteroid after a starting dose of prednisone of 0.5–1.0 mg/kg/day may reduce the development of long-term autoimmune sequelae [22–26].

These case reports support the notion that paediatricians should be aware of the clinical presentations of and therapeutic approaches for DRESS syndrome, especially in children receiving long-term antibiotic treatment. A detailed medication history is essential to achieving a diagnosis. Furthermore, RegiSCAR is a simple and reliable instrument for confirming a clinical suspicion of DRESS. The removal of the offending drug is crucial and may be the only life-saving measure. In more aggressive cases, corticosteroid or other immunosuppressive drugs should be considered to achieve the best outcome.

## Abbreviations

CRP: C-reactive protein
DHR: Drug hypersensitivity reactions
DRESS: Drug reaction with eosinophilia and systemic symptoms
INR: International normalized ratio
LDH: Lactate dehydrogenase
RegiSCAR: Register of Severe Cutaneous Adverse Reactions
TARC: Serum thymus and activation-regulated chemokine
